# Characterizing human subchondral bone properties using near-infrared (NIR) spectroscopy

**DOI:** 10.1038/s41598-018-27786-3

**Published:** 2018-06-27

**Authors:** Isaac O. Afara, Cristina Florea, Ismail A. Olumegbon, Chibuzor T. Eneh, Markus K. H. Malo, Rami K. Korhonen, Juha Töyräs

**Affiliations:** 1 Department of Applied Physics, University of Eastern Finland, Kuopio, Finland; 2 Diagnostic Imaging Centre, Kuopio University Hospital, Kuopio, Finland; 3 The South Savo Social and Health Care Authority, Mikkeli Central Hospital, Mikkeli, Finland

## Abstract

Degenerative joint conditions are often characterized by changes in articular cartilage and subchondral bone properties. These changes are often associated with subchondral plate thickness and trabecular bone morphology. Thus, evaluating subchondral bone integrity could provide essential insights for diagnosis of joint pathologies. This study investigates the potential of optical spectroscopy for characterizing human subchondral bone properties. Osteochondral samples (n = 50) were extracted from human cadaver knees (n = 13) at four anatomical locations and subjected to NIR spectroscopy. The samples were then imaged using micro-computed tomography to determine subchondral bone morphometric properties, including: plate thickness (Sb.Th), trabecular thickness (Tb.Th), volume fraction (BV/TV), and structure model index (SMI). The relationship between the subchondral bone properties and spectral data in the 1^st^ (650–950 nm), 2^nd^ (1100–1350 nm) and 3^rd^ (1600–1870 nm) optical windows were investigated using partial least squares (PLS) regression multivariate technique. Significant correlations (p < 0.0001) and relatively low prediction errors were obtained between spectral data in the 1^st^ optical window and Sb.Th (R^2^ = 92.3%, error = 7.1%), Tb.Th (R^2^ = 88.4%, error = 6.7%), BV/TV (R^2^ = 83%, error = 9.8%) and SMI (R^2^ = 79.7%, error = 10.8%). Thus, NIR spectroscopy in the 1^st^ tissue optical window is capable of characterizing and estimating subchondral bone properties, and can potentially be adapted during arthroscopy.

## Introduction

Osteoarthritis (OA) is a condition of synovial joints often associated with pain, cartilage erosion, immobility and general joint dysfunction. Although extensive research on the pathogenesis of OA have focused primarily on mechanisms involved in the destruction of articular cartilage, studies^1–3^ have suggested and demonstrated that changes in the underlying subchondral bone properties may be key indicators in the initiation and progression of OA. For example, thickening of the subchondral bone plate has been shown to be an important clinical manifestation in OA patients. This is because the health of the overlying cartilage depends on the mechanical integrity of the subchondral bone, which affects the load-bearing capability of the overlying cartilage^1^. Furthermore, it has been argued that certain types of primary OA in humans are initiated from the subchondral bone, rather than disease related to the cartilage matrix^4^. In addition, certain animal models of OA suggest that subchondral bone thickening occurs earlier than any visible changes in cartilage^5^. These observations highlight the importance of characterizing subchondral bone integrity for intra-operative decisions during treatment.

Currently, subchondral bone changes during the development of OA are detected and quantified using a variety of biomarkers of bone formation^6^ and resorption^7,8^. Since this approach is indirect and anatomically non-specific, clinical diagnosis of subchondral bone changes and remodelling rely heavily on clinical radiographic examination, which allows detection of subchondral bone sclerosis and joint space narrowing. However, this method is unreliable for detecting early-stage subchondral bone microstructural changes, which may precede the appearance of cartilage lesions. More so, visual assessment during arthroscopic surgery does not provide information on subchondral bone integrity. Thus, there is need for diagnostic methods that can penetrate through the cartilage layer and detect subchondral bone changes in OA, with potential for *in vivo* application. In this study, we propose the use of near infrared (NIR) spectroscopy and investigate its capacity for accomplishing this task.

NIR spectroscopy is a vibrational spectroscopic technique that is sensitive to molecular species containing specific bonds (C–H, N–H, O–H, and S–H) that characterize the fundamental structures of biological materials. In addition, the reduced photon absorption in the visible-NIR spectral range (650–2500 nm) allows deeper penetration into soft tissues^9,10^, enabling *in vivo* evaluation of soft tissues. The visible-NIR spectral region consists of three main tissue optical windows: the first optical window^11^ (650–950 nm) which includes part of the visible and the short-NIR region, the second^11,12^ (1100–1350 nm) and third optical^13^ (1600–1870 nm) windows, which encompass the longer-wavelength NIR bands.

The capacity of NIR spectroscopy to non-destructively characterize connective tissues has been previously shown in the literature^14–21^, with only one study demonstrating the capacity of this optical method to assess subchondral bone integrity in an animal model of OA^22^. Other vibrational spectroscopy methods, such as Raman^23^ and mid-IR^24,25^ spectroscopy, have also been proposed for assessment of bone integrity. Mid-IR is restricted by poor penetration depth into soft tissue (~10 µm into the overlaying articular cartilage), while the capacity of Spatially Offset Raman Spectroscopy (SORS)^23^ for assessment of subchondral bone integrity through the overlying cartilage has not been investigated. However, penetration through soft tissues is a unique feature of NIR spectroscopy^9,10^, making it an ideal technique for deep-chondral and subchondral tissue assessment. In this study, we investigate the potential of NIR spectral data in the three optical windows to assess human subchondral bone properties, based on the hypothesis that light in the different optical windows penetrate to different depths of osteochondral tissues and could provide diagnostic information related to the subchondral bone properties. To test this hypothesis, we investigate the relationship between the spectral response of human osteochondral samples in the different optical windows and their subchondral bone properties using partial least squares regression (PLSR) analytical technique, augmented with spectral pre-processing. The subchondral bone parameters, including subchondral bone plate thickness (Sb.Th), trabecular bone thickness (Tb.Th), bone volume fraction (BV/TV) and structure model index (SMI) were obtained using micro computed tomography (micro-CT).

## Methodology

### Sample preparation

**Figure 1:**
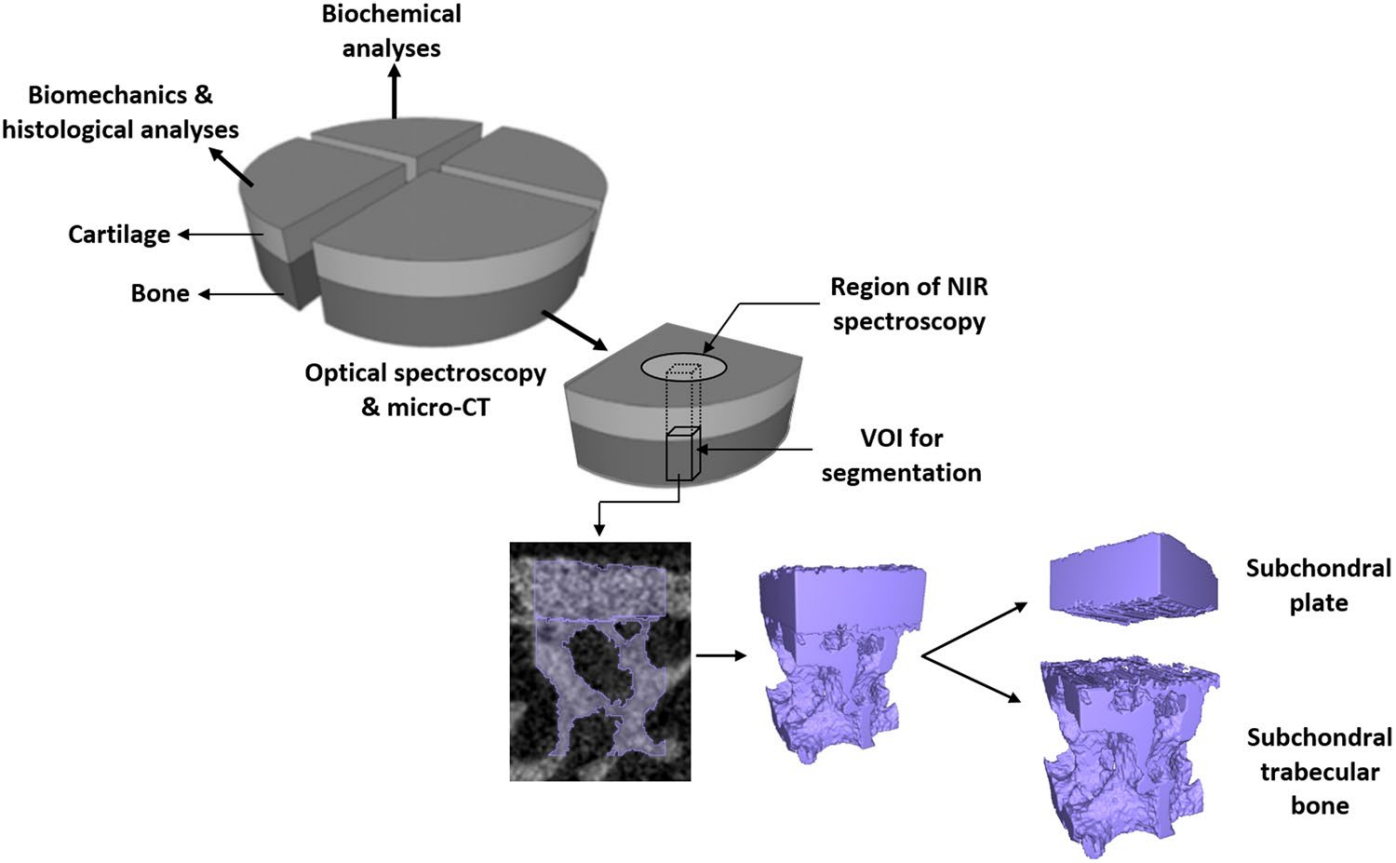
Overview of the study protocol, showing sample preparation and experimental methodologies. Cylindrical osteochondral samples (dia. = 16 mm) were obtained from human cadaver knee joints and divided into quadrants. One quadrant was subjected to NIR spectroscopy via a fibre optic probe (window dia. = 2 mm) and then imaged with micro-CT. Volume of interest (VOI: 1 × 1.1 × 1.8 mm^3^) within the region subjected to NIR spectroscopy was selected for image segmentation to obtain microstructural subchondral bone properties.

Cylindrical osteochondral specimens (n = 50, dia. = 16 mm) were drilled from the knee joints of human cadavers (N = 13, 12 males, 1 female; 29–76 years old, mean age = 53.5) with no known history of joint diseases at four anatomical locations^26^, namely: femoral lateral condyle (FLC, n = 12), femoral groove (FG, n = 12), tibial medial plateau (TMP, n = 13), and tibial lateral plateau (TLP, n = 13). Two samples (each from FLC and FG) were excluded due to almost complete loss of cartilage. Informed consent was not obtained since the samples were obtained from human cadaver subjects. The specimens were divided into quadrants which were subjected to different test protocols, only one of the quadrants was used in this study (Fig. 1).

The knee joints were obtained from Jyväskylä Central Hospital, Jyväskylä, Finland under ethical approval by the National Authority for Medicolegal Affairs, Helsinki, Finland (Permission 1781/32/200/01). The experiment and protocols were performed in accordance with relevant guidelines and regulations of the aforementioned authority. After extraction, the samples were immersed in phosphate-buffered saline (PBS) containing inhibitors of proteolytic enzymes (ethylenediaminetetraacetic acid dehydrate— EDTA; Merck, Darmstadt, Germany) and benzamidine HCl (Sigma, St. Louis, MO, USA) and then frozen. Prior to experiments, the samples were thawed and immersed in PBS supplemented with inhibitors of proteolytic enzymes throughout the study. The samples were only thawed prior to testing in order to minimize the number of freeze-thaw cycles.

### Near infrared (NIR) spectroscopy

Diffuse reflectance NIR spectroscopy of the samples was performed using an Avantes spectrometer (wavelength 200–2500 nm, AvaSpec-ULS2048XL, Avantes BW, Apeldoorn, Netherlands) and light source (wavelength 360–2500 nm, power 5 W, optical power 239 µW in 600 µm fiber, Avantes BW, Apeldoorn, Netherlands). The spectrometer is equipped with a custom-designed fibre optic probe (dia. = 5 mm) consisting of seven fibres (dia._fibre_ = 600 µm) within the central window (dia. = 2 mm). Six of the fibres were used for transmitting the NIR light, and the central fibre for collecting the diffuse reflected light from the sample. Data acquisition and monitoring were performed on a personal computer running Avasoft 8.0 software (Avantes BW, Apeldoorn, Netherlands).

Prior to spectral acquisition from the samples, dark spectra were collected with the light source off to eliminate environmental factors such as stray light. Subsequently, a reference spectrum was acquired from a 99% reflectance standard with the fibre optic probe perpendicular and in contact with the reflectance standard. The absorbance spectrum was calculated from the diffuse reflectance, dark and reference spectra as stated in our previous studies^20,27^. Three spectral measurements were taken per sample, with probe realignment prior to each measurement. Each spectral measurement consisted of 10 co-added scans, and the final spectrum was calculated as the average of the three repetitions. It was also ensured that micro-CT analyses were conducted within the region of each sample that was subjected to NIR spectroscopy.

### Micro-CT characterization of human subchondral bone properties

After NIR measurements, the samples were imaged using a high-resolution cone-beam micro-CT scanner (Skyscan 1172, Bruker micro-CT, Kontich, Belgium) with an isotropic voxel size of 12.5 μm and a 0.5 mm thick aluminium filter. The X-ray tube voltage was set to be 100 kV and the current was 100 μA. The X-ray projections were obtained at 0.4° rotation step with 316 ms exposure time. The cross-sectional images were reconstructed using a modified Feldkamp cone-beam algorithm (NRecon software, v.1.6.2.0, Skyscan, Bruker micro-CT, Kontich, Belgium). The reconstructed micro-CT data was first imported into Mimics software (v.19, Materialise, Belgium) for visualization of the three-dimensional (3D) geometry of the osteochondral samples and segmentation.

To distinguish bone tissue from cartilage, all reconstructed grayscale images were segmented, using a fixed global threshold defined by visual inspection of the segmentation result. Subsequently, a 1.0 × 1.1 × 1.8 mm^3^ volume of interest (VOI) was placed in the subchondral bone, within the region of the sample where spectral measurements were obtained **(**Fig. 1**)**. The VOIs containing both subchondral plate and subchondral trabecular bone were then manually segmented by two independent investigators using a contour-based tool in Mimics, in order to delineate the two components **(**Fig. 1**)**. The subchondral plate and trabecular bone were segmented manually a few voxels away from the endocortical boundary using a previously described criterion based on the size of intracortical pores^28,29^. According to this criterion, the endocortical boundary splits the pore in the case of large pores, whereas the pore was included in the subchondral plate region if the size of a pore was less than twice the average size of pores in that region or if the pore size was smaller than the distance from the pore to the endosteal region (i.e., the border between bone and bone marrow).

Subsequently, the segmented subchondral bone plate and trabecular bone masks were exported using seg3D software^30^. Three dimensional (3D) morphometric parameters of subchondral bone were then calculated using CTAn software (Skyscan, v.1.13, Bruker micro-CT, Kontich, Belgium) according to the American Society for Bone and Mineral Research (ASBMR) guidelines^31^. The 3D morphometric parameters obtained include: subchondral plate thickness (Sb.Th); trabecular thickness (Tb.Th), describing the mean thickness of the trabeculae; trabecular bone volume fraction (BV/TV), describing the ratio of bone volume to tissue volume; and structure model index (SMI), a quantification of the trabecular bone structure as either rod- or plate-like structure. The subchondral bone parameters were determined from the mean value of the segmentation results of the independent investigators. Since it is possible for dislodged bone particles from the trabecular bone to get stuck in the marrow of extracted osteochondral plugs during drilling, a smaller VOI within the central portion of each sample was analysed in order to minimise the potential effect of bone particle artefacts on the morphometric parameters.

### Evaluation of cartilage integrity

Articular cartilage integrity was evaluated histologically from Safranin-O stained tissue sections obtained from the samples as described in our previous study^26^. Tissue integrity was assessed by three independent assessors using the conventional Mankin scoring system^32^. The samples were blind-coded and three sections were scored per sample by each assessor. The final score was obtained as the average of the scores from all assessors, rounded to the nearest integer^26^. Although human cadaver joints with no known history of joint diseases were used in this study, some of the samples exhibited signs of degeneration, possibly due to aging. To evaluate the relationship between cartilage integrity and subchondral bone properties, the samples were classified into two groups: the first group (Class 1) consisted of samples with Mankin score <4, while the second (Class 2) included samples with Mankin score >4. In the context of this study, ‘Class 1’ represents normal samples and samples with ‘early’ stage degeneration, while ‘Class 2’ represents samples with ‘mild to advanced’ degeneration.

### Statistical evaluation

Statistical analyses of the subchondral bone morphological parameters were performed in Graphpad Prism (version 5.0). The data were expressed as mean ± 95% CI (confidence interval). Test for normality was performed for samples obtained from each anatomical location using D’Agostino and Pearson omnibus normality test, and was passed by all subchondral bone parameters, except for BV/TV. Thus, difference between samples from each anatomical location was compared using one-way ANOVA, a *p*-value of less than 0.05 was considered to be statistically significant. Kruskal-Wallis non-parametric statistical method was employed for comparison of differences in BV/TV between the different anatomical locations. Difference in subchondral bone parameters between the two groups defined based on Mankin score (Class 1 and 2) was performed using *t*-test. In the case of lack of normality, the non-parametric Mann-Whitney’s test was employed.

### Multivariate Analyses: Partial Least Squares Regression

The samples’ spectral data (predictor/dependent variables) were correlated with their reference subchondral bone properties (independent variables) using partial least squares regression (PLSR). This bilinear regression technique extracts factors from the predictors (NIR data) and regresses them to the reference variable (subchondral bone properties)^33^, with the factors formed on the basis of maximizing covariance between the dependent and independent variables. This method is more efficient than other multivariate techniques, such as principal component regression and multiple linear regression, for analysis of spectral data. The regression (calibration) models developed are then validated to obtain the accuracy and performance of each model when used for predicting the reference variables from predictors of unknown samples. In summary, knowledge of the sample qualities (i.e. reference properties) together with the spectral data is used to derive an empirical predictive model.

Prior to PLSR analysis, non-linearities such as those ensuing from light scattering variations in reflectance spectroscopy^34^ are often corrected/linearized using pre-processing algorithms including multiplicative scatter correction (MSC) and derivative pre-treatment. To investigate the potential of NIR spectroscopy for estimating the underlying subchondral bone integrity, data from the three tissue optical windows were individually considered in the analyses. Each region was separately pre-processed and correlated with the subchondral bone morphological properties of the samples using the single **y**-variable PLSR algorithm (PLS1). k-fold (k = 10) cross-validation method was used in the validation process to determine the optimal number of PLSR factors for each model, and to estimate the performance of the calibration models. To select the best model, while potentially minimizing under- and over-fitting, optimal model selection was based on minimizing the number of PLSR components and root mean square error of cross-validation (RMSECV), while maximizing the coefficient of determination (*R*^2^). Multivariate analyses were performed using MATLAB R2016b (Natick, MA, USA).

## Results

**Figure 2:**
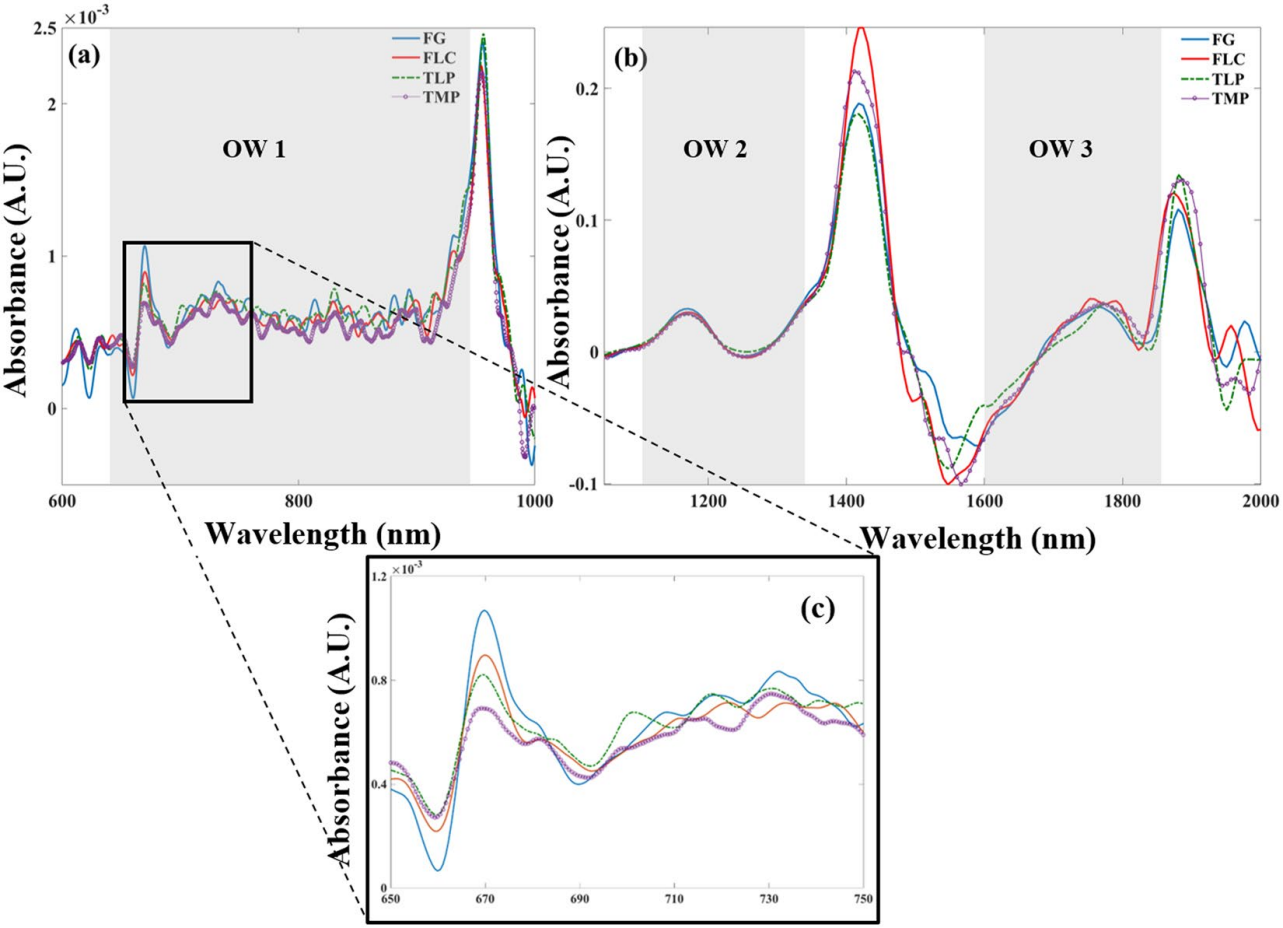
Representative 1^st^ derivative NIR spectra of samples from the different anatomical locations highlighting the (**a**) 1^st^ (OW 1), (**b**) 2^nd^ (OW 2) and 3^rd^ (OW 3) optical windows. The close-up view of a region of OW 1 (**c**) shows spectral variation with anatomical location consistent with the trend of differences in subchondral bone properties between FLC, TLP and TMP. [FG = femoral grove; FLC = femoral lateral condyle; TLP = tibial lateral plateau; TMP = tibial medial plateau].

**Figure 3:**
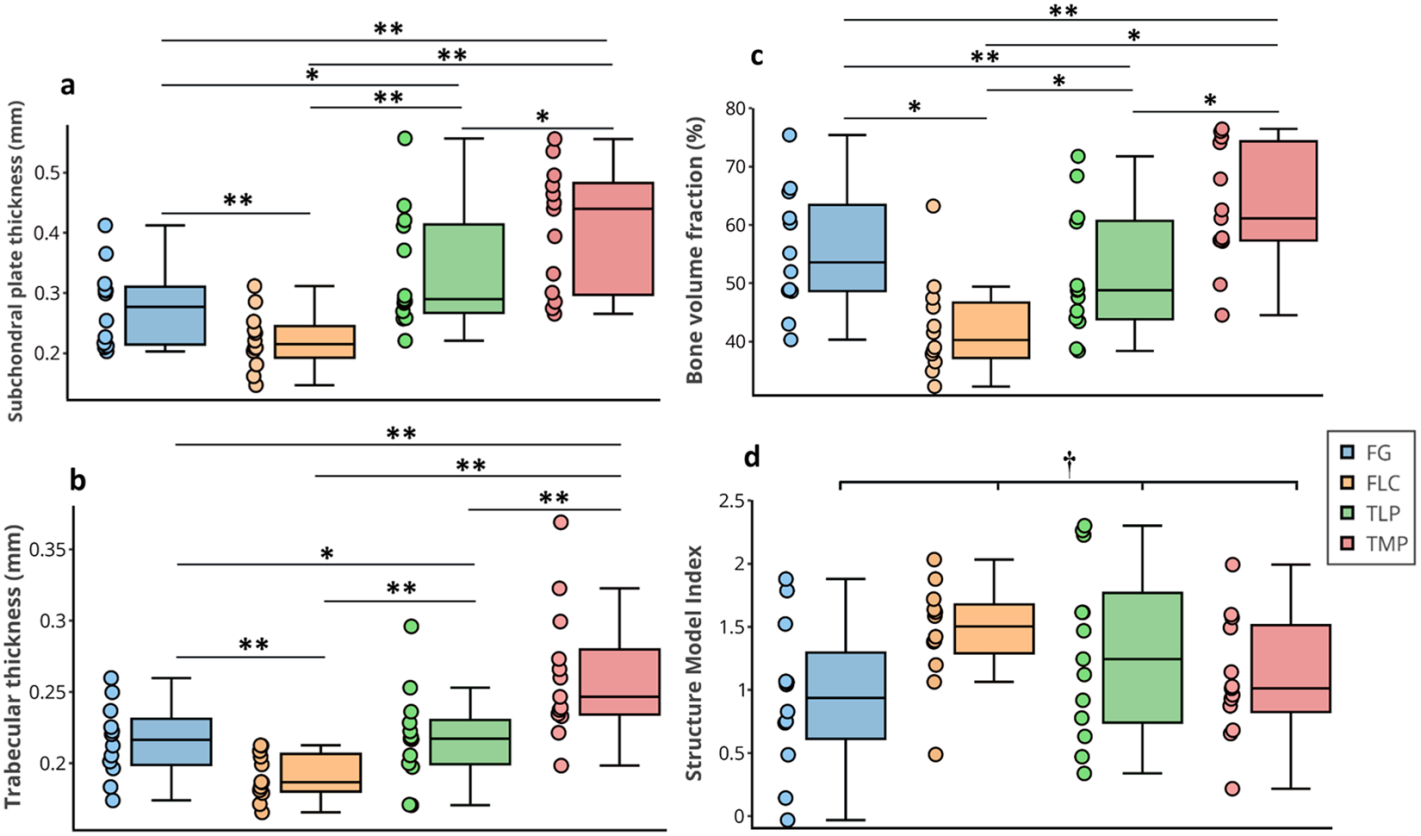
Box plot showing comparison of subchondral bone morphometric parameters from different anatomical locations of human knee joint showing differences in (**a**) subchondral plate thickness (Sb.Th), (**b**) trabecular thickness (Tb.Th), (**c**) bone volume fraction (BV/TV), and (**d**) structure model index (SMI). Values are mean ± 95% CI, **p < 0.05, *p > 0.05 and ^†^*p* = 0.117.

Representative NIR spectra of samples from the different anatomical locations within the three optical windows (Fig. 2a,b) show variations in the 1^st^ optical window consistent with the subchondral bone properties (Fig. 2c), as highlighted in the distribution of the parameters for each anatomical location (Fig. 3).

Statistically significant differences (*p* < 0.0001) were observed in the Sb.Th, Tb.Th and BV/TV of the samples from the different anatomical locations (Fig. 3a–c), with samples from the tibial medial plateau (TMP) showing consistently higher values than the other locations. Comparison of subchondral bone parameters from the different anatomical locations using *t*-test and non-parameteric Mann-Whitney’s test showed different levels of statistical significance (Table 1). However, no significant difference (*p* = 0.117) was observed between the SMI of samples from the different anatomical locations (Fig. 3d). It is worth noting that while the female subject (age = 74 years old) was postmenopausal, the subchondral bone morphometric properties from the subject indicate no significant deviation from the mean morphometric parameters of the entire group.

**Table 1:** Statistical comparison of subchondral bone parameters from the different anatomical locations. Underlined texts indicate results from non-parametric Mann-Whitney’s test.

	FG	FLC	TLP	FG	FLC	TLP
	**Sb.Th**			**Tb.Th**		
TMP	0.001	<0.001	0.045	0.006	<0.001	0.011
FG	—	0.029	0.095	—	0.001	0.846
FLC		—	0.001		—	0.018
	**BV/TV**			**SMI**		
TMP	0.089	<0.001	0.01	0.494	0.053	0.351
FG	—	0.003	0.34	—	0.022	0.165
FLC		—	0.024		—	0.531

**Figure 4:**
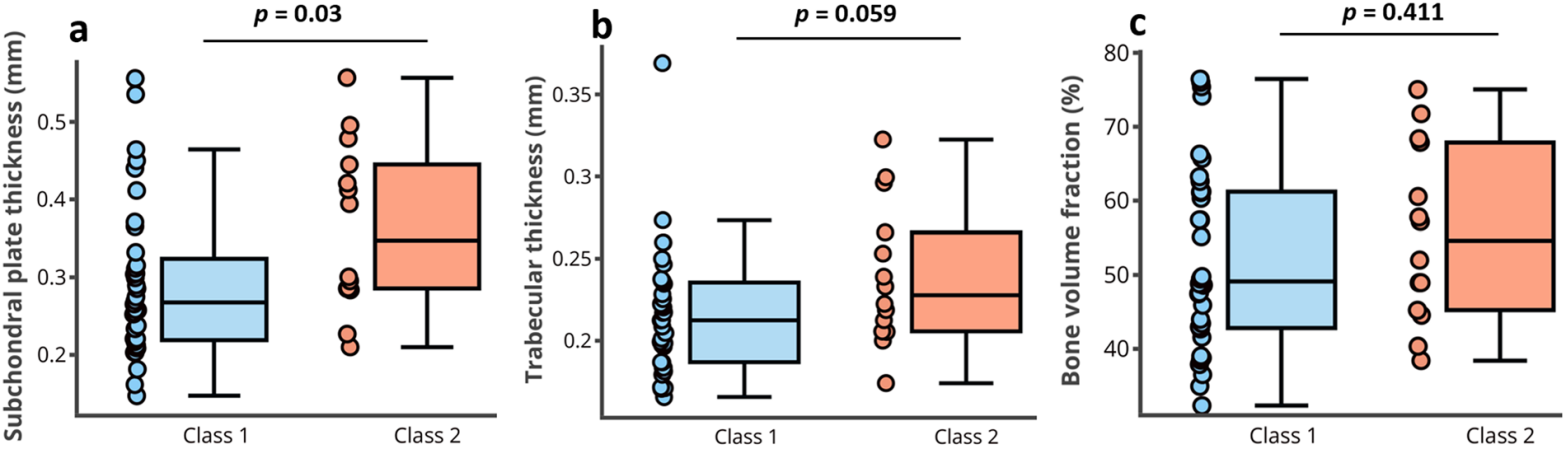
Box plot showing comparison of subchondral bone morphometric parameters of samples grouped according to cartilage integrity assessed via Mankin score. Statistically significant difference in subchondral plate thickness, Sb.Th (**a**), was observed between samples in ‘Class 1’ (Mankin score: <4, early stage degeneration) and ‘Class 2’ (Mankin score: >4, advanced degeneration). No significant difference in trabecular thickness, Tb.Th (**b**), and bone volume fraction, BV/TV (**c**) was observed between samples in both groups.

The Mankin score of the samples ranged from 1 to 9, with the following values presented as mean (lower and upper 95% CI): FG, 2.33 (1.55, 3.12); FLC, 2.1 (1.66, 2.51); TLP, 3.2 (1.92, 4.38); TMP, 3.5 ± (2.13, 4.95). The samples in ‘Class 2’ (Mankin score > 4, *i*.*e*. samples with mild to advanced degeneration) showed significantly higher Sb.Th (0.36 ± 0.06 mm, *p* = 0.03) than those in ‘Class 1′ (Mankin score < 4, *i*.*e*. normal and samples with early degeneration, 0.29 ± 0.03 mm, Fig. 4a). However, no statistically significant differences were observed in Tb.Th (*p* = 0.059, Fig. 4b) and BV/TV (*p* = 0.411, Fig. 4c) between the groups. No statistical comparison, in terms of level of degeneration, was performed for SMI because of the lack of statistically significant differences between samples from the different anatomical locations.

**Figure 5:**
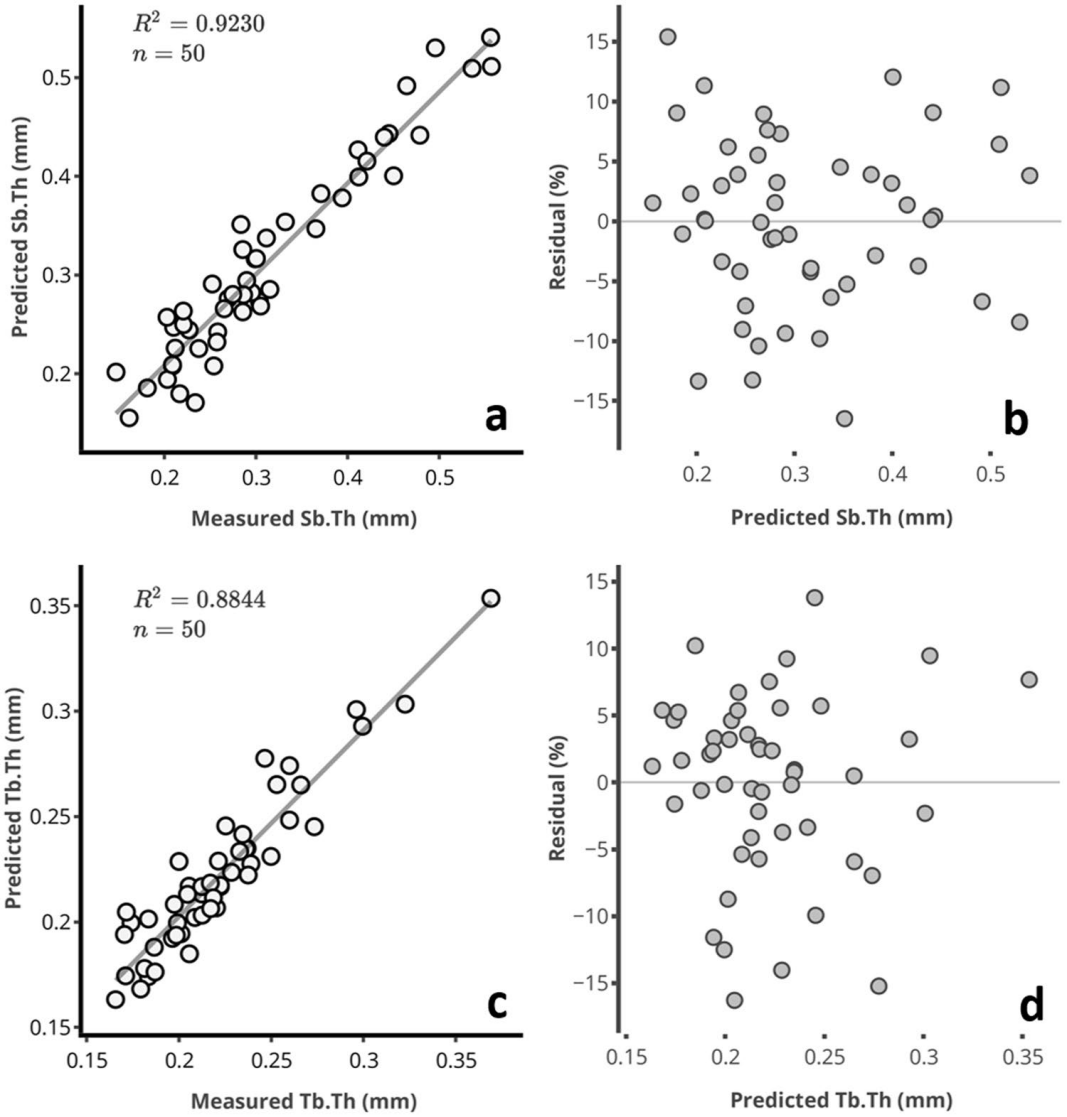
Relationship between spectral measured and predicted (**a**) subchondral plate thickness, Sb.Th, and (**c**) trabecular thickness, Tb.Th, of human osteochondral samples. The residual plots for (**b**) Sb.Th, and (**d**) Tb.Th, show the relative percentage error of prediction for each sample relative to the range.

**Figure 6:**
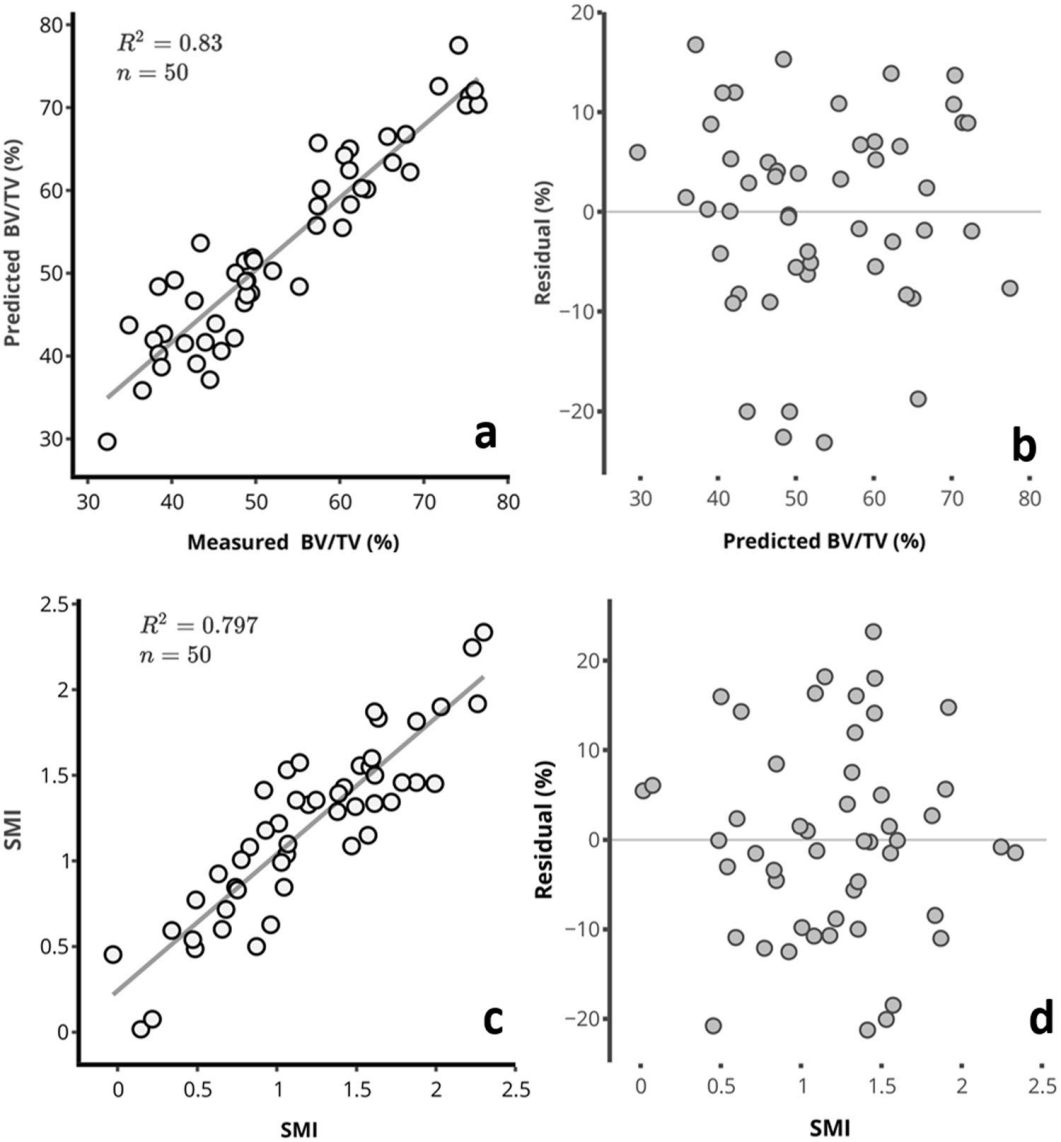
Relationship between spectral measured and predicted (**a**) bone volume fraction, BV/TV, and (**c**) structure model index, SMI, of human osteochondral samples. The residual plots for BV/TV (**b**) and SMI (**d**), show the relative percentage error of prediction for each sample relative to the range.

The relationships between the spectral data and subchondral bone parameters were optimized using 2^nd^ derivative pre-processing (Table 2) to eliminate baseline variations^35^. Spectral data from the 1^st^ tissue optical window was found to be optimal for estimating subchondral bone parameters (Table 2), with strong correlations (*R*^2^) and low relative errors obtained between the spectral-predicted and measured parameters (Figs 5 and 6). This suggests that unlike the 2^nd^ and 3^rd^ optical windows, spectral data in the 1^st^ optical window experiences minimal interference from the overlying cartilage matrix.

**Table 2:** Multivariate analysis assessment of the relationship between spectral data in the different optical windows and subchondral bone parameters.

	N	R^2^	RMSECV (%)	RMSEC (%)
	Optical window 1*			
Sb.Th	6	92.3	20.5	7.1
Tb.Th	6	88.4	16.4	6.7
BV/TV	6	83	25.2	9.8
SMI	5	79.7	22.5	10.8
	**Optical window 2**			
Sb.Th	6	43.0	25.4	19.4
Tb.Th	5	52.3	17.6	13.7
BV/TV	4	30.6	27.6	22.9
SMI	7	43.2	24.3	18.1
	**Optical window 3**			
Sb.Th	6	45.9	24.4	18.9
Tb.Th	5	48.9	18.8	14.2
BV/TV	5	65.7	28.4	20.0
SMI	7	47.7	24.5	17.4

[N = number of PLS components; *R*^2^ = coefficient of determination; RMSECV = root mean square error of cross-validation; RMSEC = root mean square error of calibration. Both error parameters are estimated relative to the range of the reference data]. *R*^2^ for BV/TV was calculated using non-parametric (Spearman) method because of the lack of normality. *Optimal tissue optical window.

The relationship between the measured and spectral-predicted subchondral bone parameters in the first tissue optical window are presented in the scatter and residual plots of Figs 5 and 6. The residual plots show the percentage error of prediction for all the samples relative to the range.

## Discussion

We assess for the first time the capacity of optical spectroscopy in specific NIR spectral regions (tissue optical windows) for non-destructive evaluation of human subchondral bone properties. The optimal spectral region for estimation of subchondral bone properties, *i*.*e*. the 1^st^ optical window, is consistent with our previous study^22^ where the capacity of optical spectroscopy for predicting subchondral bone properties in pre-clinical rat models of OA was demonstrated. The outcome of this study suggests that spectral data in the different optical windows encode information related to cartilage^19^ and subchondral bone^22^ properties. Furthermore, the results indicate that wavelength-dependent penetration of light into osteochondral samples plays a significant role in the relationship between the optical response and subchondral bone properties. This is evident in the high correlation (Table 2) obtained with data in the 1^st^ optical window, which penetrates deeper than light in the other windows. This is consistent with literature^13^, and supports our hypothesis that light in the different optical windows possess different penetration depth into osteochondral tissues that could provide information related to subchondral bone properties. Although Fourier transform infrared (FTIR) spectroscopy is capable of assessing bone fragility and fracture risk^24^, which requires bone biopsy, this study demonstrates the extension of a related optical technique, NIR spectroscopy, for non-destructive assessment of subchondral bone quality.

Based on the present results and existing literature on the capacity of NIR spectroscopy to monitor different levels of OA-related changes in cartilage^19,36^ and subchondral bone^22^, it is our position that this optical technique is capable of simultaneous characterization of articular cartilage and subchondral bone integrity in healthy and diseased human joints. This presents significant potential for augmenting conventional arthroscopy in clinical assessment of defective joints, *e*.*g*., detecting the severity and extent of joint tissue degeneration, which enables optimal diagnosis of joint condition and supports informed treatment decision.

The significant differences in Sb.Th, Tb.Th and BV/TV of samples from the different anatomical locations (Fig. 3a,b and d) are likely due to anatomical variations in joint loading. For example, the medial compartment is more prone to degeneration; thus, this could be a possible explanation for the consistently higher Sb.Th, Tb.Th and BV/TV parameters of samples from TMP. In addition, samples from this anatomical location possessed higher Mankin score than other locations, indicating higher levels of cartilage degeneration due to aging. The lack of statistically significant difference in SMI of samples from the different anatomical locations is probably due to sensitivity of this parameter to the micro-CT VOI selected, which may not have covered entire trabeculae, since SMI measures the plate- or rod-like geometry of trabecular structures^37^. It is worth noting that since the selected micro-CT VOI may not have fully covered the trabeculae within its bounding region (*i*.*e*. some portion of the trabeculae may have been ‘cut’) the SMI obtained may not have been reliably estimated by the spectra.

The significant difference in Sb.Th observed between samples in Class 1 and Class 2, *i*.*e*., early versus advanced cartilage degeneration (Fig. 4a), confirms a strong relationship and interplay between cartilage integrity and subchondral plate thickness. In this interplay, early stage cartilage degeneration results in changes in subchondral bone plate mechanical properties, resulting in increased plate thickness due to increased mineral crystallinity and non-hierarchical intra-fibrillar mineralization^38^, ultimately leading to further cartilage damage. This finding is consistent with existing studies where increase in plate thickness was observed in human^3^ and rabbit^39^ joints during OA; although the joints employed in the present study exhibited signs of cartilage degeneration and not clinically diagnosed OA. Furthermore, the increased plate thickness with overall cartilage degeneration is consistent with bone sclerosis in OA, both in humans, primates and guinea pigs^2,39,40^. This suggests modifications of bone metabolism, which has been characterized in human OA by increased cancellous bone collagen metabolism^41^.

Conversely, no significant difference in Tb.Th (Fig. 4b) and BV/TV (Fig. 4c) was observed between samples in Class 1 and Class 2. While this is contrary to the findings of Bobinac *et al*.^3^, where increased Tb.Th and BV/TV were observed in human joint samples with OA, our findings are consistent with Fahlgren *et al*.^39^, where it was suggested that the first bone response to joint degeneration occurs in the subchondral bone plate rather than in the underlying trabecular bone. More so, it is accepted that the subchondral plate and trabecular bone each respond differently and should be approached as separate structures^2,42^. Thus, this suggests that the pattern of subchondral bone modification during degeneration may not follow a linear trend with articular cartilage degeneration. In other words, visual characterization of the severity of cartilage degeneration does not provide sufficient information for estimation of the underlying subchondral bone condition^22^.

As mentioned earlier, the subjects had no known history of joint diseases; however, signs of degeneration were observed in some of the samples, likely due to aging. It is known that during aging, structural and compositional changes occur in joint tissues, including cartilage surface fibrillation^43^, altered bone turnover^44^ and increased subchondral bone density^45^, predisposing the joint to development of OA. In this study, differentiation between the levels of joint degeneration was based on cartilage integrity assessed histologically via the Mankin score, with more degenerated samples exhibiting significantly higher subchondral plate thickness, which is related to altered bone turnover. We do note however that this study aims to investigate the potential of NIR spectroscopy for characterising human subchondral bone integrity, with potential application in diagnosis of OA-related changes in the joint during arthroscopy.

The significantly linear relationship (Table 1) between the NIR-predicted and measured subchondral bone morphometric parameters (Figs 5 and 6) obtained with data from the 1^st^ optical window is possibly due to a combination of absorption and scattering of the deep penetrating light from the subchondral bone. Spectral absorption observed in the NIR spectrum of biological materials arises primarily from C–H, N–H, O–H and S–H bonds^46^, and also indicate micro- and macroscopic changes in their structure. Thus, the NIR spectrum of osteochondral samples contains latent information on physical and structural characteristics of both articular cartilage and the underlying subchondral bone^19,22^. Thus, application of spectral data from specific tissue optical windows enables extraction of depth-dependent information, as NIR light is capable of penetrating to about 8.5 mm into biological tissue^9^. The spectral absorptions observed in OW 1 are due to 3^rd^ overtone C–H and N–H^46^ vibrations associated with amide-related fundamental vibrations in the mid-IR spectral region, and arguably indicative of the matrix collagen content. It is worth noting that the bands in the NIR region are relatively wide and often overlapping, making assessment of specific peaks difficult due to their underlying anharmonic nature. This presents difficulty in separating the contributions due to collagen in cartilage and subchondral bone. However, since light in this region penetrates deeper, these vibrations are likely to arise from subchondral bone collagen, the major extra-cellular matrix component of bone^24,47^.

In addition to light penetration and scattering from within the subchondral bone, some of the light is scattered from the cartilage-bone interface and is likely a function of the interface roughness. Thus, this parameter could potentially provide information on subchondral osteophytes, and could be an indicator of OA, which triggers subchondral bone remodelling and osteophyte formation. Although, this was not investigated in the present study, the method could be extended to detection of pathological indicators of OA. The poor correlation obtained with data from the 2^nd^ and 3^rd^ optical windows is due to substantial absorption of light within the cartilage matrix^48^, since spectral measurements were performed through articular cartilage, thereby reducing the amount of NIR light reaching the subchondral bone.

A potential limitation of the present study is that the exact interaction and depth of penetration of light in the different optical windows into cartilage and subchondral bone is not fully understood. In addition, the interaction of light with the three subchondral mineralized tissues, namely calcified cartilage, subchondral cortical bone, and subchondral trabecular bone, which are distinguished morphologically, physiologically, and mechanically^42^, is not fully understood. This would require computational modelling of light transport and interaction with soft and hard tissues, which is beyond the scope of the current study. Nevertheless, this study presents a significant contribution to knowledge that could advance non-destructive evaluation of subchondral bone condition in real-time. In addition, the effect of bone marrow and its contribution to the spectra of osteochondral samples, was not accounted for in this study. This effect, we believe, can only be fully accounted for via modelling as mentioned above.

This study has demonstrated, for the first time, the potential of NIR spectroscopy to assess human subchondral bone properties as a means for rapid and non-destructive estimation of subchondral bone integrity in OA. Although this approach has been previously demonstrated in rat models of OA^22^, we present a step closer to clinical adaptation of this optical technique for arthroscopic evaluation of subchondral bone integrity. Combining spectral data in the 1^st^ tissue optical window with derivative pre-processing and multivariate analysis, NIR spectroscopy has the capacity to support *in vivo* quantitative evaluation of subchondral bone changes in defective joints. In conclusion, this optical method has the potential to facilitate real-time non-destructive arthroscopic evaluation of subchondral bone changes that occur as part of the OA development process.

## References

[1] Radin, E. L. & Rose, R. M. Role of subchondral bone in the initiation and progression of cartilage damage. *Clin*. *Orthop*. *Relat*. *Res*. 34–40 (1986). 3780104

[2] Intema F (2010). In early OA, thinning of the subchondral plate is directly related to cartilage damage: results from a canine ACLT-meniscectomy model. Osteoarthr. Cartil..

[3] Bobinac D, Spanjol J, Zoricic S, Maric I (2003). Changes in articular cartilage and subchondral bone histomorphometry in osteoarthritic knee joints in humans. Bone.

[4] Batiste DL (2004). *Ex vivo* characterization of articular cartilage and bone lesions in a rabbit ACL transection model of osteoarthritis using MRI and micro-CT 1. Osteoarthr. Cartil..

[5] Pastoureau PC, Chomel AC, Bonnet J (1999). Evidence of early subchondral bone changes in the meniscectomized guinea pig. A densitometric study using dual-energy X-ray absorptiometry subregional analysis. Osteoarthr. Cartil..

[6] Garnero P (2002). Uncoupling of type II collagen synthesis and degradation predicts progression of joint damage in patients with knee osteoarthritis. Arthritis Rheum..

[7] Garnero P (2001). Cross sectional evaluation of biochemical markers of bone, cartilage, and synovial tissue metabolism in patients with knee osteoarthritis: relations with disease activity and joint damage. Ann. Rheum. Dis..

[8] Garnero P, Rousseau J-C, Delmas PD (2000). Molecular basis and clinical use of biochemical markers of bone, cartilage, and synovium in joint diseases. Arthritis Rheum..

[9] Faris F (1991). Noninvasive *In vivo* near-Infrared Optical Measurement of the Penetration Depth in the Neonatal Head. Clin. Phys. Physiol. Meas..

[10] Afara I, Singh S, Oloyede A (2013). Application of near infrared (NIR) spectroscopy for determining the thickness of articular cartilage. Med. Eng. Phys..

[11] Anderson RR, Parrish JA (1981). The Optics of Human Skin. J. Invest. Dermatol..

[12] Smith AM, Mancini MC, Nie S (2009). Bioimaging: Second window for *in vivo* imaging. Nat. Nanotechnol..

[13] Sordillo LA, Pu Y, Pratavieira S, Budansky Y, Alfano RR (2014). Deep optical imaging of tissue using the second and third near-infrared spectral windows. J. Biomed. Opt..

[14] Spahn G (2010). Near-infrared spectroscopy for arthroscopic evaluation of cartilage lesions: results of a blinded, prospective, interobserver study. Am. J. Sports Med..

[15] Spahn G (2008). Evaluation of cartilage defects with near-infrared spectroscopy (NIR): an *ex vivo* study. Med. Eng. Phys..

[16] Palukuru UP, McGoverin CM, Pleshko N (2014). Assessment of hyaline cartilage matrix composition using near infrared spectroscopy. Matrix Biol..

[17] McGoverin CM, Lewis K, Yang X, Bostrom MPG, Pleshko N (2014). The Contribution of Bone and Cartilage to the Near-Infrared Spectrum of Osteochondral Tissue. Appl. Spectrosc..

[18] Afara IO, Singh S, Oloyede A (2013). Load-unloading response of intact and artificially degraded articular cartilage correlated with near infrared (NIR) absorption spectra. J. Mech. Behav. Biomed. Mater..

[19] Afara I, Prasadam I, Crawford R, Xiao Y, Oloyede A (2012). Non-destructive evaluation of articular cartilage defects using near-infrared (NIR) spectroscopy in osteoarthritic rat models and its direct relation to Mankin score. Osteoarthr. Cartil..

[20] Afara IO, Hauta-Kasari M, Jurvelin JS, Oloyede A, Töyräs J (2015). Optical absorption spectra of human articular cartilage correlate with biomechanical properties, histological score and biochemical composition. Physiol. Meas..

[21] Afara IO (2014). Near infrared spectroscopy for rapid determination of Mankin score components: a potential tool for quantitative characterization of articular cartilage at surgery. Arthroscopy.

[22] Afara I, Prasadam I, Crawford R, Xiao Y, Oloyede A (2013). Near infrared (NIR) absorption spectra correlates with subchondral bone micro-CT parameters in osteoarthritic rat models. Bone.

[23] Buckley, K. *et al*. Measurement of abnormal bone composition *in vivo* using noninvasive Raman spectroscopy. *IBMS Bonekey* **11**, (2014).

[24] Gourion-Arsiquaud S (2009). Use of FTIR Spectroscopic Imaging to Identify Parameters Associated With Fragility Fracture. J. Bone Miner. Res..

[25] Boskey A, Pleshko N (2007). FT-IR imaging of native and tissue-engineered bone and cartilage. Biomaterials.

[26] Kurkijärvi JE, Nissi MJ, Kiviranta I, Jurvelin JS, Nieminen MT (2004). Delayed gadolinium-enhanced MRI of cartilage (dGEMRIC) and T2 characteristics of human knee articular cartilage: topographical variation and relationships to mechanical properties. Magn. Reson. Med..

[27] Sarin JK (2016). Near Infrared Spectroscopic Mapping of Functional Properties of Equine Articular Cartilage. Ann. Biomed. Eng..

[28] Malo MKH (2013). Longitudinal elastic properties and porosity of cortical bone tissue vary with age in human proximal femur. Bone.

[29] Florea C (2015). Alterations in subchondral bone plate, trabecular bone and articular cartilage properties of rabbit femoral condyles at 4 weeks after anterior cruciate ligament transection. Osteoarthr. Cartil..

[30] University of Utah SCI. Seg3D: Volumetric Image Segmentation and Visualization. (2010).

[31] Parfitt AM (2009). Bone histomorphometry: Standardization of nomenclature, symbols, and units: Report of the asbmr histomorphometry nomenclature committee. J. Bone Miner. Res..

[32] Mankin HJ, Dorfman H, Lippiello L, Zarins A (1971). Biochemical and metabolic abnormalities in articular cartilage from osteo-arthritic human hips. II. Correlation of morphology with biochemical and metabolic data. J. Bone Jt. Surg. Am.

[33] Bjørsvik, H.-R. & Martens, H. Data analysis: Calibration of NIR instruments by PLS regression. in *Handbook of Near Infrared* Analysis (eds. Burns, A. D. & Ciurczak, E. W.) 185–207 (Marcell Dekker, Inc., 2001).

[34] Geladi P, MacDougall D, Martens H (1985). Linearization and Scatter-Correction for Near-Infrared Reflectance Spectra of Meat. Appl. Spectrosc..

[35] Brown CD, Vega-Montoto L, Wentzell PD (2000). Derivative Preprocessing and Optimal Corrections for Baseline Drift in Multivariate Calibration. Appl. Spectrosc..

[36] Afara IO, Prasadam I, Arabshahi Z, Xiao Y, Oloyede A (2017). Monitoring osteoarthritis progression using near infrared (NIR) spectroscopy. Sci. Rep..

[37] Hildebrand T, Rüegsegger P (1997). Quantification of Bone Microarchitecture with the Structure Model Index. Comput. Methods Biomech. Biomed. Engin..

[38] Zuo Q (2016). Characterization of nano-structural and nano-mechanical properties of osteoarthritic subchondral bone. BMC Musculoskelet. Disord..

[39] Fahlgren A, Messner K, Aspenberg P (2003). Meniscectomy leads to an early increase in subchondral bone plate thickness in the rabbit knee. Acta Orthop. Scand..

[40] Hayami T (2006). Characterization of articular cartilage and subchondral bone changes in the rat anterior cruciate ligament transection and meniscectomized models of osteoarthritis. Bone.

[41] Mansell JP, Bailey AJ (1998). Abnormal cancellous bone collagen metabolism in osteoarthritis. J. Clin. Invest..

[42] Burr DB (2004). Anatomy and physiology of the mineralized tissues: role in the pathogenesis of osteoarthrosis. Osteoarthr. Cartil..

[43] Martin JA, Buckwalter JA (2002). Aging, articular cartilage chondrocyte senscence and osteoarthritis. Biogerontology..

[44] Loeser RF (2010). Age-related changes in the musculoskeletal system and the development of osteoarthritis. Clinics in Geriatric Medicine.

[45] Dingemanse, W. *et al*. A prospective follow up of age related changes in the subchondral bone density of the talus of healthy Labrador Retrievers. *BMC Vet Res*. **13**, (2017). 10.1186/s12917-017-0974-y PMC5319136 28219379

[46] Stuart, B. Organic Molecules. in *Infrared spectroscopy: fundamentals and applications* 86–87 (John Wiley & Sons, Ltd, 2004).

[47] Wen X-X (2015). Time Related Changes of Mineral and Collagen and Their Roles in Cortical Bone Mechanics of Ovariectomized Rabbits. PLoS One.

[48] Padalkar MV, Pleshko N (2015). Wavelength-dependent penetration depth of near infrared radiation into cartilage. Analyst.

